# Selective Breeding for Voluntary Wheel-Running Behavior in Mice is not Associated with Reduced Lifetime Reproductive Success or Lifespan Under Long-Term Paired Breeding Conditions

**DOI:** 10.1007/s10519-026-10277-x

**Published:** 2026-08-12

**Authors:** Natalie N. Whitehead, Nicole E. Schwartz, Chenkun Jiang, Haley M. Read, Natalie C. Holt, Theodore Garland

**Affiliations:** 1 Department of Evolution, Ecology, and Organismal Biology, University of California, Riverside, CA 92521 USA; 2 Present Address: Department of Biology, Pomona College, Claremont, CA 91711 USA

**Keywords:** Costs of reproduction, Genetic drift, Lifespan, Reproductive success, Trade-off, Voluntary exercise

## Abstract

We studied lifespan and lifetime reproductive success (LRS) in mice from a selection experiment that includes 4 replicate High Runner (HR) lines bred for voluntary wheel-running behavior and 4 non-selected Control (C) lines. HR mice typically run ~ threefold farther on a daily basis, leading to substantially increased energy expenditure and food consumption, and are also more active and eat more when housed without wheels. HR and C mice have been shown to differ for many other traits that could affect reproduction, including body mass and composition, aerobic capacity, hormonal profile (including corticosterone and leptin), and other behavioral traits (e.g., maternal care, thermoregulatory nesting, anxiety-like behavior in an elevated plus maze). At generation 97, male–female pairs were maintained until end of life in standard cages without wheels, with ad lib food and water. The number of pups weaned (LRS) ranged from 0–80, with a mean of 29.1 ± 8.1 (SE) for C lines and 25.8 ± 4.4 for HR (P = 0.73). Thus, artificial selection for high voluntary exercise did not significantly alter LRS. In addition, we found no statistical difference in average lifespan for dams or sires. However, replicate lines differed significantly for several traits, presumably due primarily to random genetic drift. At the level of individual variation, correlations among reproductive traits and lifespan provided little evidence for negative associations, and LRS was not negatively related to parental longevity. Overall, long-term selection on voluntary locomotor behavior did not produce detectable reductions in reproductive output or lifespan under standard laboratory conditions.

**Supplementary Information:**

The online version contains supplementary material available at https://doi.org/10.1007/s10519-026-10277-x.

## Introduction

Locomotor behavior is a complex and highly integrated phenotype that reflects interactions among neural motivation systems, musculoskeletal function, metabolic capacity, and endocrine regulation. Because locomotion involves coordinated functioning of multiple physiological systems, selection on locomotor behavior may be expected to produce correlated responses in other life history traits owing to pleiotropic gene action that results in genetic correlations. Artificial selection experiments provide a direct approach for evaluating such potential correlated responses to selection. Here, we test for correlated changes in life history traits in a long-term selection experiment that targeted voluntary wheel-running behavior (Swallow et al. 1998).

The High Runner mouse selection experiment includes 4 replicate lines of HR mice that have been bred for > 100 generations for voluntary locomotor behavior on wheels and an additional 4 lines that serve as non-selected Controls (C) (Swallow et al. 1998). Since reaching selection limits at around generation 17–27 (Careau et al. 2013), the HR mice have been running ~ threefold farther on a daily basis as compared with C mice, for both sexes, which leads to substantially increased energy expenditure and food consumption (Rezende et al. 2009). When housed without wheels, HR mice are still more active than C in their home cages and eat more than C mice, although the difference is not as great as with wheels (Copes et al. 2015; Hiramatsu and Garland 2018). In principle, this higher energetic cost of physical activity might lead to trade-offs with other costly endeavors, such as reproduction (be see Cohen et al. 2019).

Various other behavioral differences have been observed between the HR and C lines, including for maternal care (when housed with wheels) (Schwartz et al. 2025), building thermoregulatory nests (Carter et al. 2000), and anxiety-like behavior in an elevated plus maze (Singleton and Garland 2019). These behaviors and activity levels share neurobiological substrates and biochemical pathways to some degree, which might lead to trade-offs (Garland et al. 2022). Differences in maternal care and in nest building have obvious relevance for reproduction, but differences in anxiety-like behavior may also be relevant. For example, cage cleaning is on a weekly schedule, and if that happens when a litter is young, then the inherent level of anxiety could affect the likelihood of litter neglect and eventual loss. In general, demonstrated differences between HR and C mice in motivational systems, especially dopamine signaling, which has been interpreted as part of the increased motivation for wheel running (Gammie et al. 2003; Rhodes et al. 2005; Belke and Garland 2007; Saul et al. 2017; Thompson et al. 2018; Phan et al. 2024), are likely relevant to other motivated behaviors, such as maternal care.

Other characteristics of HR mice could also be relevant for reproductive abilities. For example, HR mice of both sexes are smaller in both body length and mass, and generally have less body fat (Swallow et al. 2001; Kelly et al. 2006; Hiramatsu and Garland 2018). These differences may be part of the suite of adaptive traits that support endurance running (e.g., human marathoners tend to be relatively small), but could also conflict with litter size or the size of individual offspring at birth. As would be expected, HR mice also have elevated endurance and maximal oxygen consumption (VO_2_max, adjusted for body size) during forced treadmill exercise (Meek et al. 2009; Schwartz et al. 2023). At lower levels of biological organization, HR mice have various characteristics that presumably support their higher endurance and aerobic capacity, including larger hearts and altered muscle and tendon properties (Bilodeau et al. 2009; Castro et al. 2024; Schwartz and Garland 2024). Such traits may also be relevant for sustained energy budgets that are elevated during reproduction, at least for females.

With respect to reproductive performance itself, previous studies have found little evidence for differences, although all studies only allowed a single mating and were at earlier generations (for further details, see Sect. ‘‘Discussion’’). For example, when the HR lines were nearing selection limits (Careau et al. 2013), neither litter size nor total litter mass (nor mean pup mass) at birth (generation 20) or weaning (generation 21 and 22) differed significantly between HR and C lines (Girard et al. 2002; Khan et al. 2024). Keeney (2011) compared various characteristics of both the dams and litters over several generations (40, 42, 44, 45, 47, 48, 52, and 56). For generation 44, dam estrous cyclicity, the viability of embryo-uterine implantation, postpartum maternal behaviors and litter morality assessment, pup retrieval behaviors by the dam, and the presence of pup milk spots 3–5 days were also examined (Keeney 2011). HR and C mice did not differ statistically in litter size, mass, sex ratio, length of time from dam pairing to birth of first litter, nor in measures of birth and weaning success. Approximately equal percentages of HR and C dams gave birth and for those that did give birth, equal percentages weaned at least one pup (Keeney 2011). However, when only the number of dams that weaned at least one pup was considered (as opposed to the total number of dams paired), generations 40 and 42 did show a significant reduction for HR dams for this measure of weaning success, though the difference was driven by two of the four HR lines. In addition, the duration of estrus was significantly longer in HR females, which could potentially lead to longer interbirth intervals if females were allowed to reproduce continuously. At generation 72, considering only dams that received standard chow (as in the present study), with dam mass as a covariate, HR dams had significantly larger litter sizes at weaning than C dams, but pups tended to be smaller, such that total litter mass was unaffected (Hiramatsu et al. 2017). At generation 84, HR and C dams housed without wheels (as in the present study) did not differ in maternal care behavior, birth success, weaning success, litter size or sex ratio at weaning of their offspring (Schwartz et al. 2025).

Only two studies have compared lifespans of HR and C mice, and both were incomplete in one way or another. A study at generation 16 found complicated patterns of lifespan differences between singly housed HR and C males and females in relation to housing with or without wheels (Morgan et al. 2003; Bronikowski et al. 2006). However, about half of the total 320 mice were euthanized for tissue sampling at 84 weeks of age and the experiment ended when the remaining animals were 138 weeks old (to provide for studies of aged tissues), at which time treatment groups had only 1–2 animals remaining. Thus, the power to detect differences in median lifespan expectancy were reduced and age at death could not be analyzed directly. At generation 31, a study of singly housed males did not include C mice housed without wheels as an experimental group and mean lifespan did not significantly vary among groups (Vaanholt et al. 2010).

The first goal of the present study was to measure lifetime reproductive success (LRS) of HR and C mice by housing pairs for their entire lives. To our knowledge, such a study has not previously been published for rodents of any kind. (We did not allow wheel access due to concerns over pup safety.) Our second goal was to compare lifespans of the paired males and females. Finally, we examine relationships among life history traits and test for possible costs of reproduction. Our overarching hypothesis was that HR mice would have reduced reproductive output and lifespan. Even if activity did not trade-off with LRS or lifespan due to energy limitation (given that we provided ad libitum food), changes in other components of the life history still might occur owing to shared biochemical pathways and potential functional conflicts (see above and Garland et al. 2022).

## Materials and Methods

All procedures were approved by the Institutional Animal Care and Use Committee at the University of California, Riverside.

### The High Runner Mouse Selection Experiment

The *Mus domesticus* used here were from four replicate High Runner (HR) lines that have been selectively bred for voluntary wheel-running behavior as young adults, and their four non-selected Control (C) lines. The selection experiment began with a base population of 224 outbred Hsd:ICR strain (Swallow et al. 1998). Further details are provided in the Introduction and in many previous papers (Swallow et al. 1998, 2009; Rhodes et al. 2005; Careau et al. 2013; Khan et al. 2024). Briefly, the selection criterion was total revolutions run on days five and six of a six day exposure to a Wahman type activity wheel with a 1.12 m circumference (Lafayette Instruments, Lafayette, IN, USA) when mice were ~ 6 to 8 weeks of age. These later days of testing were used to get beyond any initial period of neophobia that may occur when mice are initially exposed to a new object (e.g., see Cowan 1977; Kimball and Lattin 2024 and references therein).

Litter size was typically around 10 in earlier generations, and within-family selection was applied, as follows. For the HR lines, the highest-running female and male from each of ~ 10 litters were selected to become breeders. These siblings, representing approximately the top 20% (2 of ~ 10 full siblings per litter) based on their wheel running, would then be mated with individuals from outside their family but within their line (i.e., no sib-mating was allowed). For the C lines, wheel running was tested but no selection was applied; in other words, a male and female were chosen randomly from each family, without regard to their wheel running, and again sib-mating was disallowed. Typically, 10 breeding pairs provided offspring for the next generation. At weaning, mice were toe clipped for permanent identification and then housed 4/cage by sex.

### Experimental Design

Mice used here were sampled from generation 97, and were born between 10 and 16 October 2021, weaned at 21 days of age, and housed 4/cage by sex in standard laboratory cages with daily checks and routine health monitoring in accordance with institutional animal care guidelines. (The last mouse died on 18 April 2024.) These mice did not receive the standard 6 days of wheel access. The colony room was maintained at a room temperature of approximately 70 ° F and a relative humidity averaging approximately 45%, with ad libitum food and water, and a 12:12 light–dark cycle (07:00–19:00).

At 7 weeks of age (sexual maturity), 12 male–female pairs per line (16 in HR line 6, which is polymorphic for the mini-muscle phenotype (Kelly et al. 2013; Castro et al. 2024)) were formed by choosing at random 1 male and 1 female from each family litter while ensuring no sibling pairs were formed), and housed in standard cages with ad lib food [Teklad Rodent Diet (W) 8604, as has been used throughout the course of the selection experiment] and water.

Cages were checked daily for new births and each ensuing litter was weaned, counted, and sexed at 21 days of age. When overlapping litters occurred (which was rare: see Sect. ‘‘Results’’), they were not separated until the older one was due to be weaned. Sire-dam aggression towards pups or towards each other was not measured as these events were outside the scope of the experimental design. However, in instances where aggression occurred between sires and dams, the pair were separated for one week to allow treatment before being rehoused together if possible. Although it was inferred that some pups may have been cannibalized by parents, this was not formally assessed.

The primary outcome variable was the number of pups per pair at weaning, summed across all litters, thus yielding lifetime reproductive success. Additionally, for breeding pairs, various other life history traits (components of LRS) were evaluated, including their age at the birth of the first and last litter, number of litters born, numbers of litters and pups weaned (some litters did not have any pups survive to weaning), sex ratio at weaning (proportion female based on the total number of pups weaned). Finally, we calculated the number of litters with at least one pup weaned by the number of litters born, which should be positively related to amount or quality or parental care, although it would also reflect pups that were "non-viable," born dead or dying shortly after birth. We also recorded lifespan (age at death) of each parent and noted whether it died naturally or had to be euthanized to avoid suffering, based on veterinary examination. Aside from euthanasia, we did not attempt to ascertain the causes of death in any systematic fashion, as that was beyond the scope of this study. Common causes of death in laboratory mouse colonies include age-related neoplasias, such as lymphomas and mammary tumors (which are especially common in outbred ICR CD1 stocks), neonatal pre-weaning losses linked to maternal age, litter overlap and husbandry or social stress, infectious or subclinical pathogens, trauma and skin infections, and facility- or supplier-specific factors that affect baseline mortality (Baker 1998; Giknis and Clifford 2005; Bradley et al. 2011; Snyder et al. 2016; Morello et al. 2020). No mice died from accidental causes, such as running out of food or water.

### Statistical Analyses

Analyses were conducted using SPSS (version 30, SPSS Inc., Chicago, IL, USA), SAS 9.1 (SAS Institute Inc., Cary, NC, USA), and R (https://www.R-project.org/). As in many previous studies of these lines, a mixed-model was used to test the effects of linetype (HR vs. C mice) on the life history traits using SAS Procedure Mixed with REML estimation and Type III tests of fixed effects (e.g., Whitehead et al. 2023; Khan et al. 2024; Castro et al. 2024; Thompson et al. 2024). Replicate line was treated as a random effect nested within linetype, and the effect of linetype was tested relative to the among-line variance with 1 and 6 degrees of freedom. We also allowed for different levels of variation among the replicate HR and C lines (Garland et al. 2011). For age at death (lifespan), we had separate values for the males (sires) and females (dams), so a two-way mixed model was used. Here, the degrees of freedom for testing the effects of linetype, sex, and their interaction were also 1 and 6, and we also allowed for different levels of variation among the replicate HR and C lines.

The importance of testing for effects of such early-life factors as the size of the litter at the time an individual is weaned has recently been emphasized for rodent studies (Parra-Vargas et al. 2023). Therefore, models were fitted with and without covariates relevant to early-life conditions experienced by the parents, including the litter size that each dam or sire was weaned into and its own body mass at weaning, and results of both types of models are presented. Although these possible effects are not the main point of the present paper, they are nonetheless worth reporting for this growing area of research. In addition, we tested for an effect of cause of death for the dam or sire, which was either natural or from euthanasia following instructions from the campus veterinarian.

Statistical power to detect differences among groups can be increased by simultaneous analysis of multiple traits. Therefore, we also implemented multivariate analysis of variance (MANOVA) with SAS Procedure GLM, specifying line nested within linetype as the error term for hypothesis testing. Analyses with various combinations of dependent variables (e.g., number of litters born, LRS, ages of parents at death) never indicated a rejection of the null hypothesis, so those results are not presented.

In addition, separate analyses with SAS Procedure Mixed were conducted for HR and C lines to assess among-line variation in lifespan and life history traits. Line effects within each linetype were evaluated using separate models, treating line as a fixed effect, with degrees of freedom determined by the number of lines (four) and individuals (12–16) in each line. Again, models were run with and without covariates, including cause of death (see above). For age at death, we also performed a two-way analysis, testing for effects of sex, line, and their interaction (separately for HR and C lines).

All statistical tests were two-tailed, with significance set at α ≤ 0.05. Results are presented as least squares means (LSM) and associated standard errors (SE) from SAS Procedure Mixed. For the linetype comparisons, we intended to use the positive false discovery rate (pFDR) to control for multiple comparisons (SAS Procedure MULTTEST). However, the distribution of P values did not pass diagnostics (see Sect. ‘‘Results’’).

We used Pearson correlations and principal components analysis (PCA) to examine relationships among life history traits, both for all mice combined and separately for the two linetypes. We did not do this separately for the individual lines because of the relatively small within-line sample sizes. PCA did not seem informative, so we do not present those results. Instead, we used SAS Procedure CALIS to test the null hypothesis of no overall difference in the correlation matrices between the HR and C lines, ignoring the nested nature of the replicate lines within the two linetypes due to limitations of the software. SAS Procedure CALIS also provided tests for the differences of each individual pairwise correlation between life history traits (uncorrected for multiple comparisons).

We used non-parametric Log Rank (Mantel-Cox) tests to compare the survival distributions among the four replicate Control lines and four replicate HR lines for both dams and sires (SAS Procedure Lifetest). We did not compare overall survival curves (i.e., the cumulative number of deaths over time) statistically between the HR and C lines owing to a lack methods that properly account for the nesting of replicate lines as a random effect within linetype.

For mortality analysis, we used the R package “flexsurv” and its customization feature to fit our data with the following models that describe increasing mortality with age: Gompertz, Gompertz-Makeham, Logistics, Logistics-Makeham, and Weibull. Instantaneous hazard rate or instantaneous mortality rate (${\upmu}_{\mathrm{x}}$) represents the probability of death over a given time period. The Gompertz model for the instantaneous mortality rate is given by${\upmu}_{\mathrm{x}}=\uplambda {\mathrm{e}}^{\upgamma \mathrm{x}}$, where *λ* represents the initial mortality rate, and *γ* represents the rate of exponential increase with age. The logistic model (${\upmu}_{\mathrm{x}}=\frac{\uplambda {\mathrm{e}}^{\upgamma \mathrm{x}}}{1+\mathrm{s}\left(\frac{\uplambda }{\upgamma }\right)\left({\mathrm{e}}^{\upgamma \mathrm{x}}-1\right)}$) has an additional parameter (*s*) that represents the deceleration in the increase in mortality at the end of life; if *s* = *0*, logistic = Gompertz. Both Gompertz and Logistic models were combined with a constant additive term of early adulthood mortality (Makeham term, *c*) to obtain Gompertz-Makeham and Logistic-Makeham models. The Weibull function of mortality model (${\upmu}_{\mathrm{x}}=\uplambda + \upalpha {x}^{\beta }$) has two other parameters, *α* and*β*. *β* is a dimensionless parameter, characterizing the shape of the curve relating mortality rate and age; *α* determines the magnitude of the mortality rate at any given age for a particular value of*β*. Models were fitted for each of the four groups noted above. Coefficient estimates, model-based mortality rate estimates, loglikelihoods, and AICc of the models were obtained. For further information, see Pletcher (1999) and Ricklefs and Scheuerlein (2002). In preliminary analyses with 100-day intervals, overall, the Gompertz model provided the best fits and so those results are presented.

## Results

### Reproductive Characteristics

We first consider the reproductive outcomes for all mice in the study. Ninety-eight of the 100 breeding pairs produced at least one litter. Considering the first litter for each pair, 83 weaned at least one pup, and 89 weaned one or more pups from at least one litter (see Table 1 and Online Resource 1). Some pairs had overlapping litters or concurrent pregnancy and lactation, meaning that a new litter was born while a previous litter was still being nursed (i.e., had not yet been weaned at 21 days of age). However, such incidences of concurrent pregnancy and lactation were relatively rare and lasted at most two days (see Online Resource 2 for full details). For example, of the 92 pairs that produced a second litter, three gave birth before the previous litter was weaned (resulting in two litters in the cage at once), and three gave birth on the same day the previous litter was weaned. Considering births of the third litter for each pair (N = 83), they occurred on the same day that the previous litter was weaned for three pairs and on the day before weaning for three additional pairs.

As pairs were checked for new births only once per day, the opportunity to observe birth complications when they were happening was limited. We did not specifically record any apparent birth complications, but such instances were rarely observed, occurring in only a handful of cases.

### Comparisons of HR and Control Lines

**Fig. 1:**
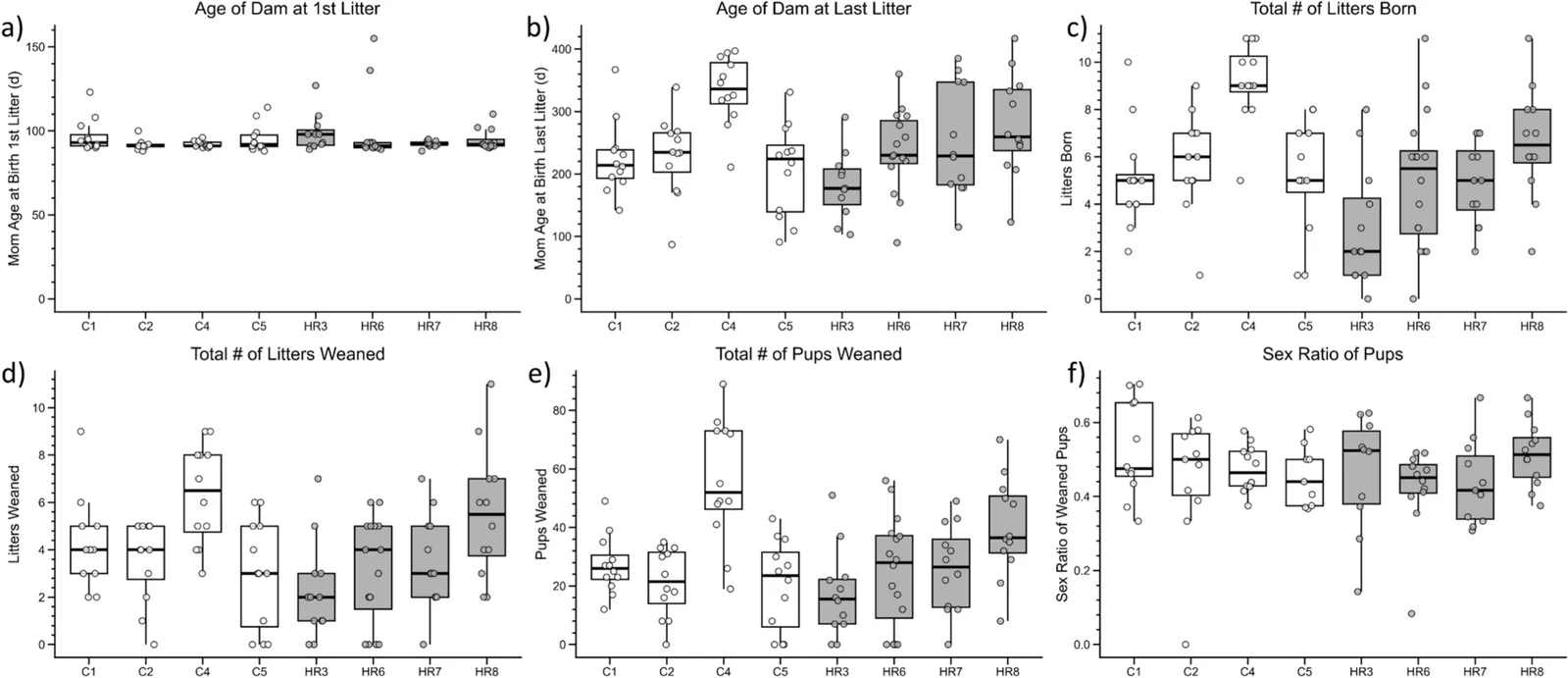
Lifetime reproductive traits for replicate High Runner and Control lines of mice. Box plots show median (middle line), first and third quartiles (Q1 bottom and Q3 top lines), and whiskers to indicate lower limit (Q1–1.5*interquartile range) and upper limit (Q3 + 1.5*interquartile range). Based on nested mixed models in SAS Procedure Mixed of data (sometimes transformed, see Table 1), no statistically significant differences were detected when comparing the four replicate Control lines with the four replicate High Runner lines for any of the traits shown here: **a** age of mother at birth of first litter (which was analyzed statistically after rank transformation), **b** age of mother at birth of last litter, **c** total number of litters born, **d** total number of litters weaned, **e** total number of pups weaned (our measure of lifetime reproductive success, or LRS), and **f** sex ratio of pups at weaning (females/total). However, significant variation (nominal P ≤ 0.05) was observed among the four replicate Control lines and among the four HR lines (ANOVAs in SAS Procedure Mixed) for all traits shown here, except pup sex ratio **f** (see Table 1). The four replicate non-selected Control lines (open bars) are designated as C1, C2, C4, and C5, whereas the selected High Runner lines (closed bars) are HR3, HR6, HR7, and HR8

We next asked whether LRS and it’s component traits differed between HR and Control lines. Comparisons of reproductive characteristics between the four replicate HR lines and the non-selected C lines showed no statistically significant differences for the total number of pups weaned (our measure of LRS) or its components, including age of mother at the birth of her first or last litter, length of the reproductive lifespan for birth or weaning, total number of litters born or weaned, and litters weaned/born (a measure of parental care) (Table 1, Fig. 1). In addition, the sex ratio of the total pups weaned did not significantly differ between the two linetypes (Fig. 1F). However, the interbirth intervals tended to be longer in HR lines, especially when considering the average value over all litters born (depending on covariates used, nominal P values ranged from 0.0453 to 0.0521 for average values) (SAS Procedure Mixed, Table 1, Online Resource 3).

**Table 1:**
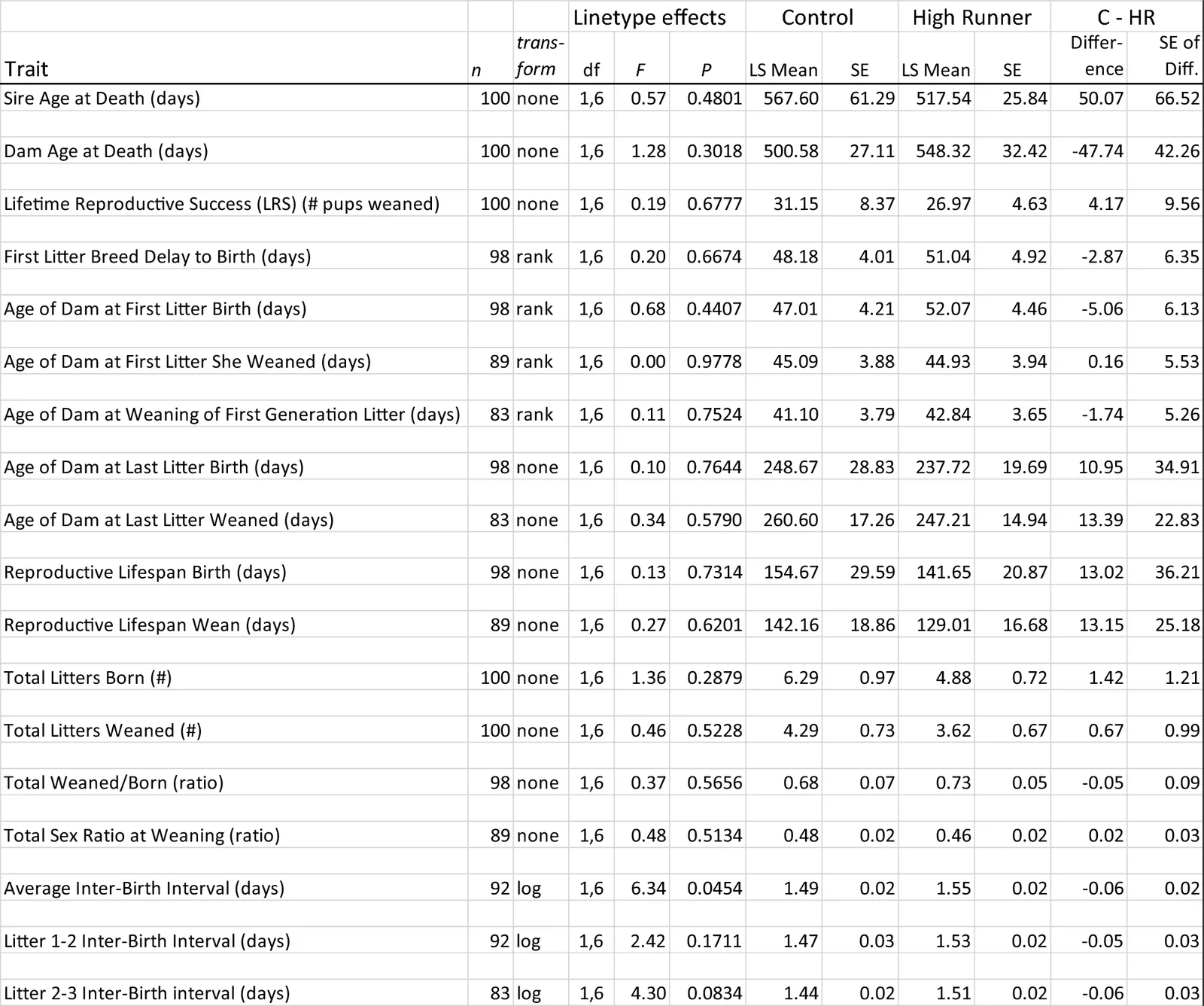
Mixed model comparisons of four selectively bred High Runner lines with four non-selected Control lines omposition of the alkaline fluorinated wastewater

HR High runner lines of mice, *n* Total sample size for the trait, *df* degrees of freedom, *F F* statistic in test of HR versus control lines, *P* Significance level for the comparison, LS Mean Least squares mean from SAS procedure mixed, *SE* Corresponding standard error of the LS mean.

We also redid all of the above analyses for linetype comparisons, but with litter characteristics of the dams, or dams and sires, and/or the cause of death of the parents, as covariates. For the full models with all eight potential parental covariates, considering the 18 the traits listed in Table 1, this yielded 162 P values (144 for the covariates plus 18 for the linetype comparisons). Nine of the covariates had nominal P < 0.05 (SAS Procedure Mixed, Online Resource 3). Based on diagnostic plots, the distribution of P values was not suitable for analysis with the positive False Discovery Rate (pFDR). The Bonferroni correction indicated that not even the smallest P value (0.0016) would be considered significant (0.0016 * 162 = 0.2621). On the other hand, considering only the litter size into which the dams and sires were weaned (Parra-Vargas et al. 2023), five have nominal P < 0.05, all of them for the sire’s litter size.

### Comparisons of the Replicate HR Lines and of the Replicate Control Lines

We then tested for variation among the replicate lines within each linetype. Various differences existed among the replicate C lines and among the HR lines (SAS Procedure Mixed, significance levels are in Online Resource 3). For the C lines, differences were found for dam age at last litter born (Fig. 1B), dam age at last litter weaned, length of reproductive lifespan for litters born and weaned, total number of litters born (Fig. 1C), total number of litters weaned (Fig. 1D), total number of pups weaned (LRS) (Fig. 1E), and the ratio of total litters weaned/total litters born. Some of the P values changed considerably when covariates were added to the models (Online Resource 3).

For the HR lines, differences were found for the age of the dam at last litter born, length of reproductive lifespan for litters born, the total number of litters born (Fig. 1C), the total number of litters weaned (Fig. 1D), and the total number of pups weaned (LRS) (Fig. 1E). Interestingly, line differences became significant for sex ratio at weaning (all litters combined) and interval between second and third litters born when the cause of death of the parents was included in the model (Online Resource 3).

Considering the four C lines, adding the litter characteristics of the dams, or dams and sires, as covariates indicated that 12 of the 162 P values were nominally < 0.05 (Online Resource 3) and one would be considered significant following the Bonferroni correction, which was for line differences in LRS (corrected P = 0.0493). When comparing the four HR lines, 15 P values were nominally < 0.05, but none would be considered significant with the Bonferroni correction.

### Lifespan

**Fig. 2:**
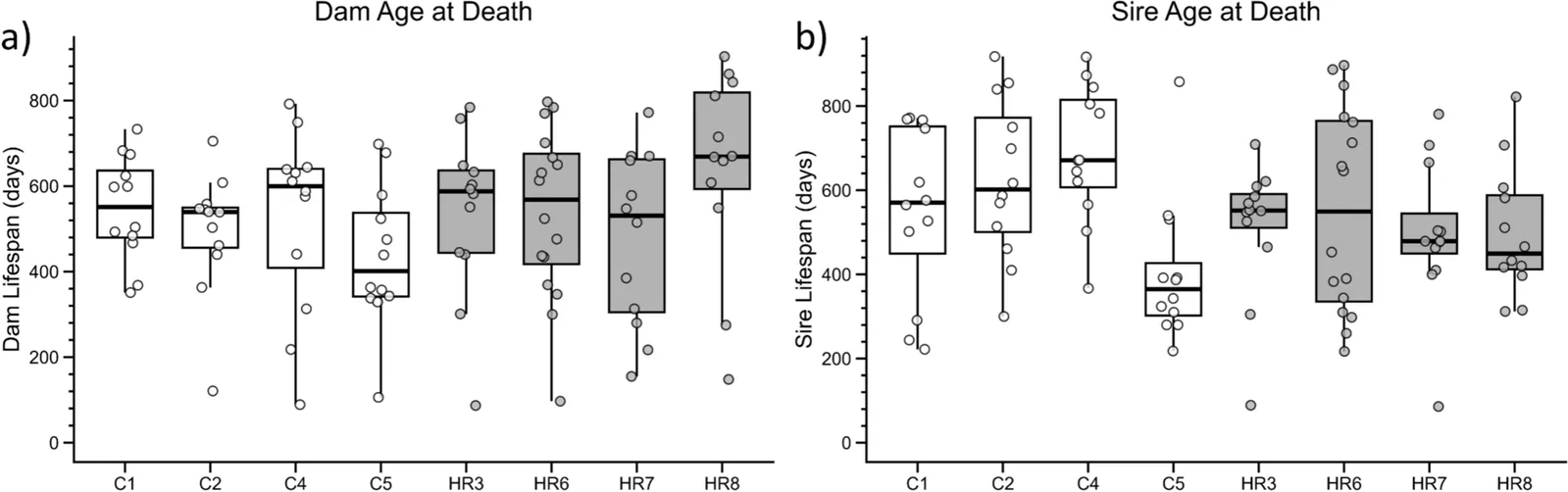
Lifespans of dams and sires across replicate High Runner and Control lines of mice. Box plots show median (middle line), first and third quartiles (Q1 bottom and Q3 top lines), and whiskers to indicate lower limit (Q1–1.5*interquartile range) and upper limit (Q3 + 1.5*interquartile range). No statistical difference in mean lifespan was detected for either **a** dams or **b** sires when comparing all four High Runner (HR) with all four non-selected Control lines (Table 1; nested mixed models in SAS Procedure Mixed). However, significant variation occurred among the replicate C lines for age of sire at death (based on a nominal P ≤ 0.05: Online Resource 3), with line C5 having a notably shorter mean lifespan (ANOVAs in SAS Procedure Mixed). Abbreviations are as indicated in Fig. 1 legend

**Fig. 3:**
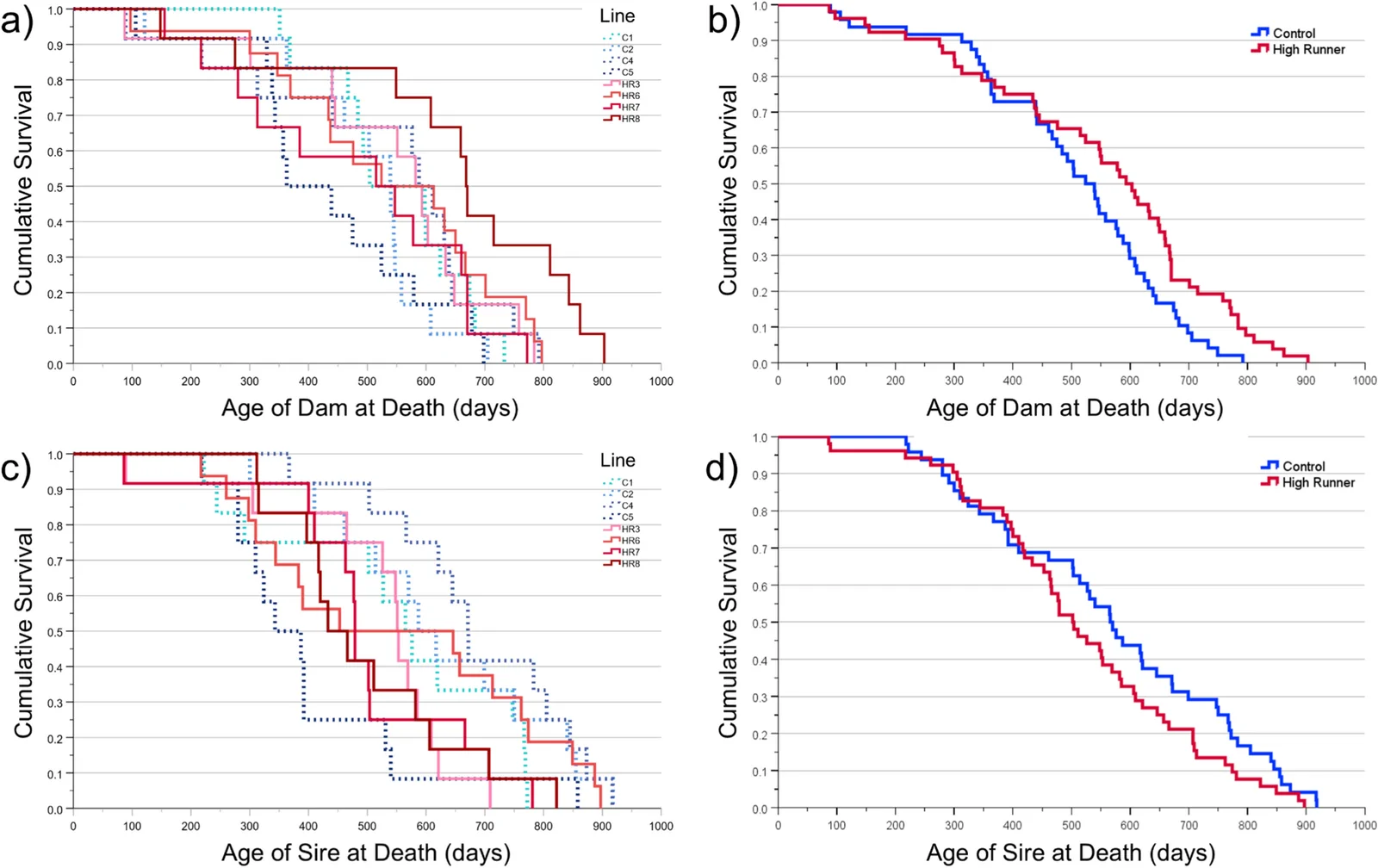
Survival of dams and sires in relation to linetype and line. Curves for HR and Control lines were not compared statistically (see Methods), but replicate lines within linetype were and showed some differences (see ‘‘Results’’)

Next, we assess whether lifespan differed between HR and Control lines, and whether early-life or experimental covariates influenced these outcomes. Table 2 provides descriptive statistics for lifespan comparisons between HR and C mice. Mean lifespan (age at death) did not differ statistically between linetypes for the mothers (SAS Procedure Mixed, P = 0.3018) or fathers (P = 0.4801) (Table 1, Fig. 2, Fig. 3). Analysis using characteristics of the litters into which the mothers and fathers had been born as covariates indicated that the father’s litter size had a positive effect his lifespan (Table 1, P = 0.01) (Online Resource 3). In contrast, the father’s litter size had a negative effect on the lifespan of his mate (i.e., the mothers) (Online Resource 3). In a two-way analysis of age at death (SAS Procedure Mixed), we found no statistical effect of sex (F_1,6_ = 0.36, P = 0.57), linetype (F_1,6_ = 0.00, P = 0.98) or their interaction (F_1,6_ = 2.59, P = 0.16).

**Table 2:**
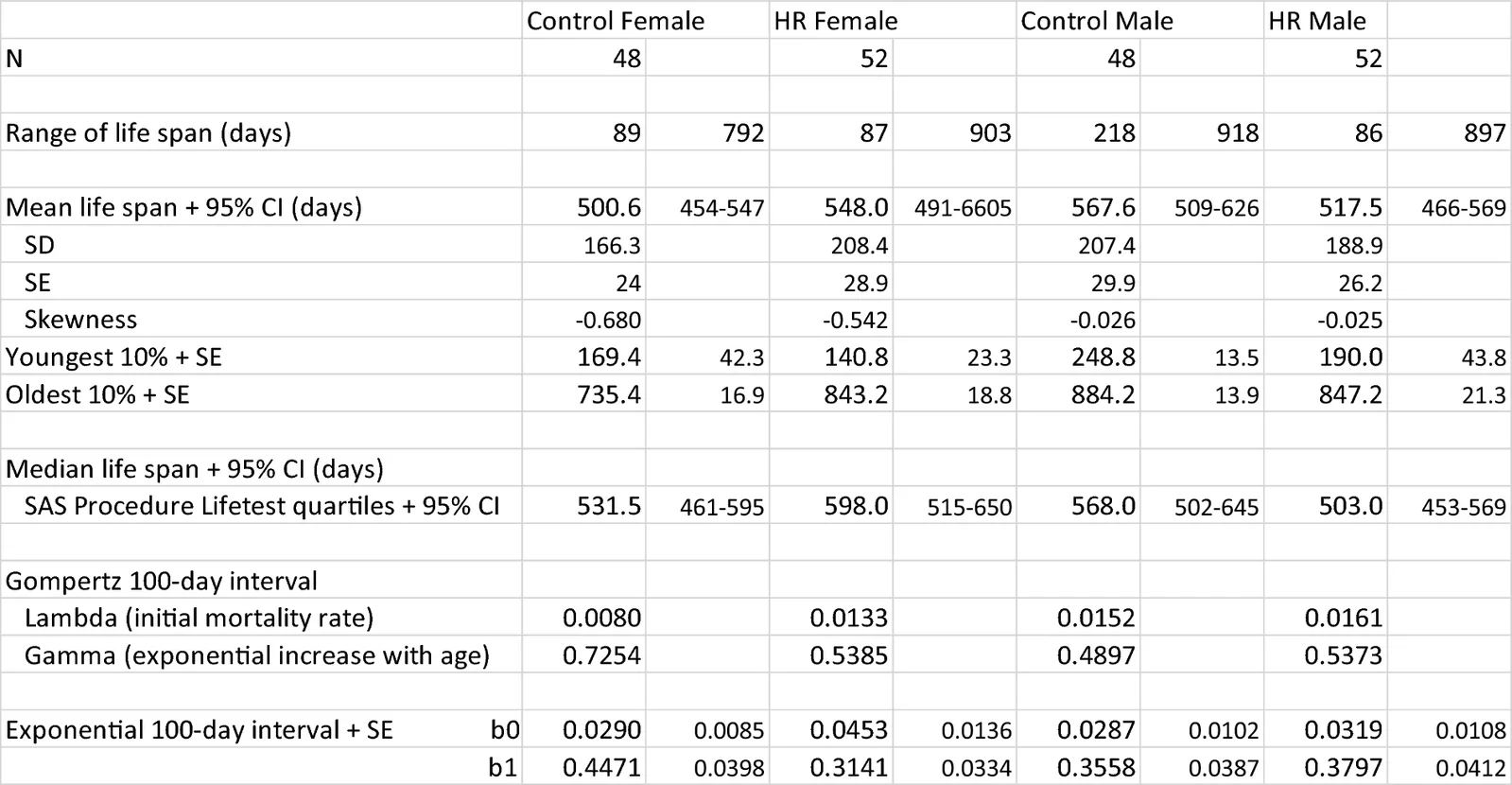
Descriptives statistics for lifespan and results of mortality analyses

The only trait compared statistically was mean lifespan, which did not statistically differ between Control and High Runner lines for either females (dams) or males (sires) (see ‘‘Results’’ section on Lifespan).

*HR* High runner lines of mice, *CI* Confidence interval, *SD* Standard deviation, *SE* Standard error of the mean, b0 and b1 are parameters in the exponential model fitted to the data.

Comparison of cause of death (frequency of euthanasia) between HR and C lines (SAS Procedure GLIMMIX) indicated no statistical difference for either dams (F_1,6_ = 0.21, P = 0.66) or sires (F_1,6_ = 0.15, P = 0.71). However, the cause of death affected dam lifespan, with those who were euthanized living longer (SAS Procedure Mixed, P < 0.002).

Based on sex-specific analyses, the replicate C lines differed for sire (SAS Procedure Mixed, P = 0.0031) but not for dam (P = 0.3912) mean lifespan, whereas the HR lines did not differ for either sire (P = 0.8499) or dam (P = 0.2855) lifespan. Adding characteristics of the litters into which the mothers and fathers had been born as covariates indicated that the father’s litter size had a positive effect on his lifespan for the HR lines (Table 1, Online Resource 3). However, for the C lines, the father’s litter size had a negative effect on the lifespan of his mate (P = 0.0302, Table 1). In a combined sex analysis of age at death for C lines (SAS Procedure Mixed), we found a trend for sires to live longer than dams (F_1,88_ = 3.52, P = 0.0639) and substantial differences among lines (F_3,88_ = 5.01, P = 0.0030), with no statistically significant interaction (F_3,88_ = 1.81, P = 0.1510). For age at death in the HR lines, we found no effect of sex (F_1,96_ = 0.75, P = 0.3883) or replicate line (F_3,96_ = 0.75, P = 0.5276), and no interaction (F_3,96_ = 0.89, P = 0.4498).

The frequency of euthanasia did not significantly differ among replicate lines for either dams or sires (SAS Procedure GLIMMIX: F_3,44_ = 1.07, P = 0.37 for Control dams, F_3,446_ = 0.55, P = 0.65 for Control sires; F_3,48_ = 1.14, P = 0.34 for HR dams, F_3,48_ = 2.39, P = 0.08 for HR sires). However, the cause of death affected dam lifespan, with those who were euthanized living longer for both HR and C lines (SAS Procedure Mixed, Online Resource 3).

### Survival Curves and Mortality Analysis

Further, we examined age-specific patterns of mortality between HR and Control. Figure 3 shows that survival was generally similar between the HR and C lines for both sexes, with some changes in survival rates around approximately 400–450 days of age. More specifically, survival rates of C females start to decline compared with those of HR females. In males, the converse occurs, with survival rates of HR mice declining relative to C mice. As a result, median lifespan (not compared statistically: see Methods) was higher in HR (598 days) than C (531.5) for females (Fig. 3b), but that pattern was reversed in males (HR = 503 days, C = 568) (Fig. 3d).

Comparisons of the survival curves for replicate lines (Fig. 3) with SAS Procedure Lifetest for females (Fig. 3a) indicated no statistical differences for C lines (log-rank *x*^2^ = 4.56, d.f. = 3, P = 0.2066), whereas HR lines showed a marginally nonsignificant difference (log-rank *x*^2^ = 7.66, d.f. = 3, P = 0.0536). For males (Fig. 3c), C lines differed (log-rank *x*^2^ = 11.30, d.f. = 3, P = 0.0102) but HR lines def. did not (log-rank *x*^2^ = 3.13, d.f. = 3, P = 0.3723).

**Fig. 4:**
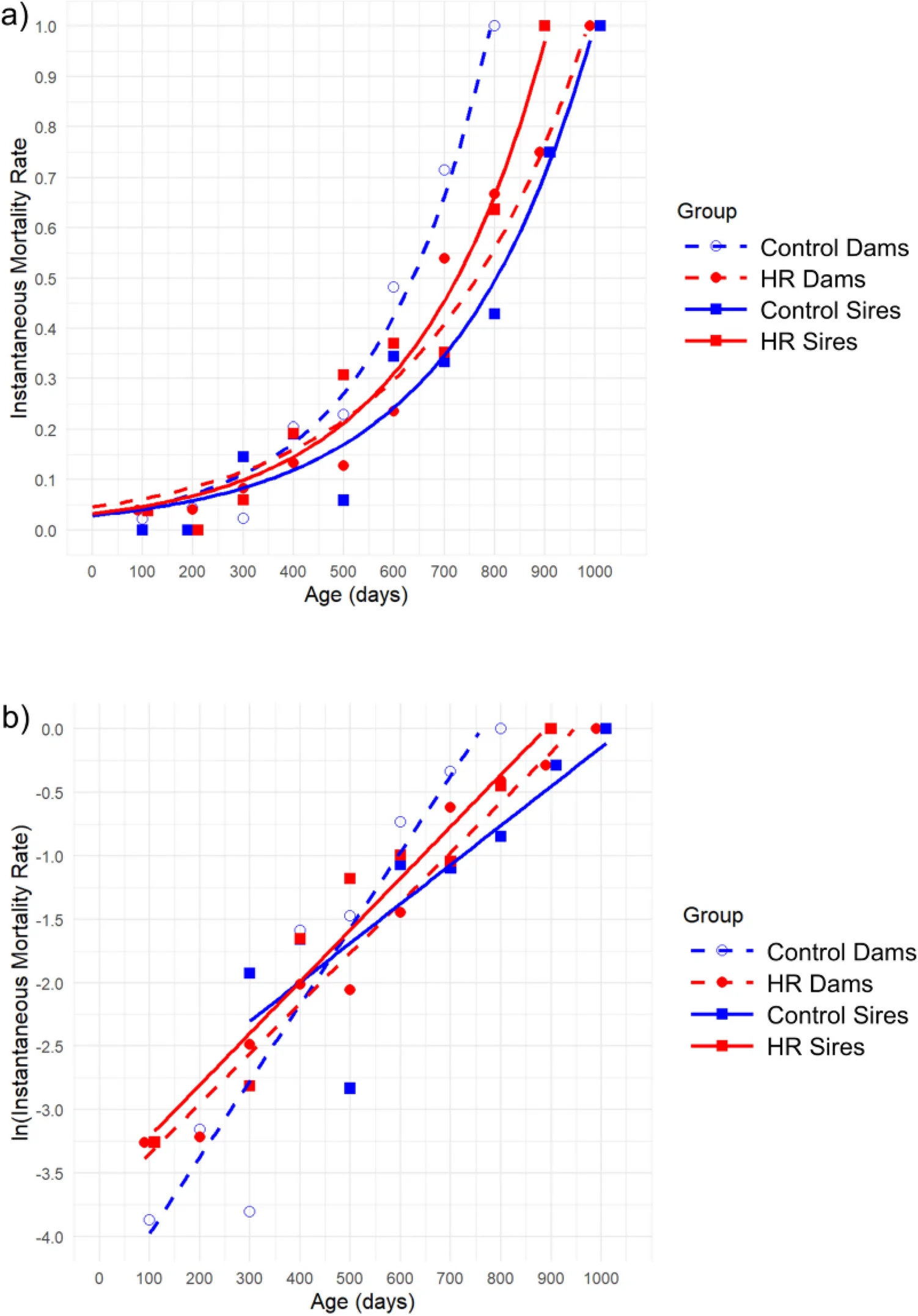
Instantaneous mortality curves by sex for replicate High Runner and Control lines of mice. **a** Instantaneous mortality rates computed for 100-day intervals and fitted with exponential curves. **b** Log_10_-transformed instantaneous mortality rates fitted with least-squares linear regressions. Note the missing values that occur when mortality is zero within a given 100-day interval (because the log of zero is undefined), which suggests caution in comparing the lines (see Sect. ‘‘Results’’)

Figure 4A shows instantaneous mortality rates in relation to age for 100-day intervals. Visually, the primary difference among groups is an increase in mortality rate in C females beginning around 400–450 days of age. Exponential fits (Table 2) describe the data reasonably well and indicate that the exponential term (± SE) for C females is somewhat larger (0.447 ± 0.040, r^2^ = 0.979) than for HR females (0.314 ± 0.033, r^2^ = 0.957), C males (0.356 ± 0.039, r^2^ = 0.956) or HR males (0.380 ± 0.041, r^2^ = 0.960). This general pattern was also obtained from Gompertz fits in R package Flexsurv, which uses all individual data points rather than mortality rates calculated for discrete intervals (Table 2). The better fit of Gompertz models as compared with logistic models (not shown, based on 100-day intervals) indicates that a late-life plateau or deceleration of mortality rates is not supported.

Figure 4B plots the natural log of instantaneous mortality rates for 100-day intervals, which results in some missing values when no mortality occurred in a given interval. For example, as can be seen in Fig. 3, no mortality occurred in C males until 218 days of age, so that group has no value shown in Fig. 4B for the first two intervals because the ln of zero is undefined. In any case, fitting a general linear model to compare the least-squares regression lines shown in Fig. 4B indicated a significant group-by-age interval interaction (P = 0.0197). Figure 4B suggests that C females have the lowest initial mortality rates, but that is misleading because C males had even lower mortality rates of zero within the first two 100-day intervals and hence missing values for those intervals.

### Relations Among Life History Traits

We examined correlations among reproductive traits, lifespan, and their components to evaluate potential life history trade-offs. Table 3 shows the correlation matrix for nine of the life history traits listed in Table 1, excluding inter-birth intervals from litters 1–2 and 2–3 to avoid a substantial drop in sample size. Considering the 98 pairs that produced at least one litter, the overall correlation matrices were quite similar. To test the null hypothesis of equivalent correlation matrices between HR and C mice, we used SAS Procedure CALIS. We first considered only the six component reproductive traits, omitting LRS and ages at death of the parents. The overall test for equality of the correlation matrices did not reject the null hypothesis of no difference between the C and HR lines (*x*^2^ = 14.50, d.f. = 15, P = 0.4878). In addition, none of the 15 correlation coefficients significantly differed (2-tailed P values ranged from 0.0603 to 0.9325). Results were similar when we added in the ages of death for the parents (*x*^2^ = 28.91, d.f. = 28, P = 0.4170). One of the 28 bivariate correlations significantly differed between HR and C mice (P = 0.0399), for the relation between age at death of the dam and mean litter size (r = 0.2230 for C mice versus −0.1695 for HR), but that would not survive any sort of correction for multiple comparisons. For both HR and C mice, the strongest correlate of LRS was the number of litters weaned (r_HR_ = 0.940, r_C_ = 0.858, both P < < 0.0001), followed by the number of litters born for HR mice (r_HR_ = 0.739, P < < 0.0001) and the mean litter size at weaning for C mice (r_C_ = 0.710, P < < 0.0001).

**Table 3:**
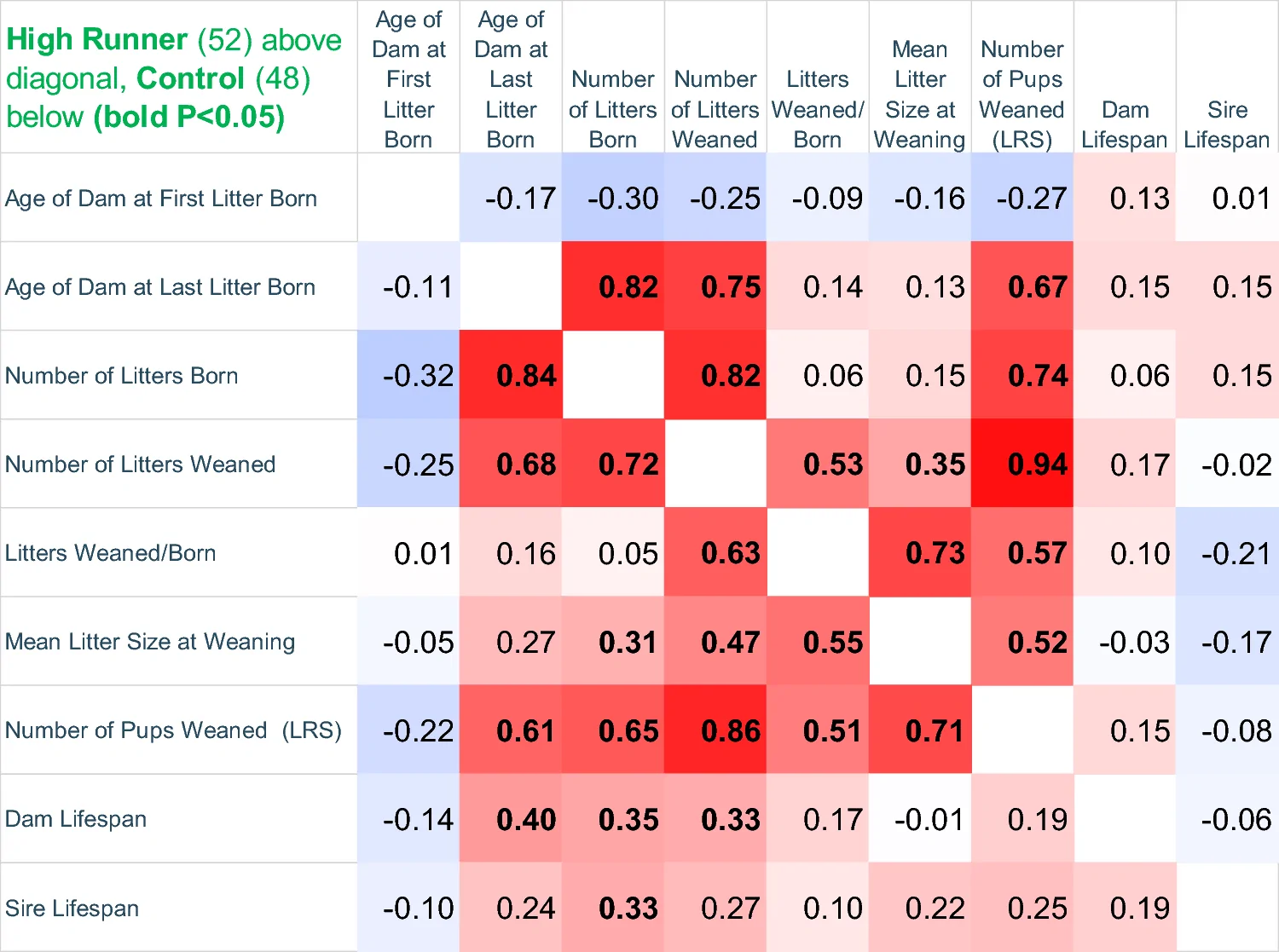
Pairwise Pearson correlations for nine life history traits

Values significant at P < 0.01 (unadjusted for multiple comparisons) are in bold.

We also tested the relationship between the size of the first litter at weaning and LRS, as the former is often used as a proxy for fitness (Powers et al. 2020). For this analysis, pairs that did not give birth to any litters (N = 2) or that did not wean their first litter (an additional 15 pairs) were assigned a value of zero for the size of the first litter. Correlations were 0.636 for all 100 pairs (P < 0.0001), 0.666 for the 48 C pairs (P < 0.0001), and 0.660 for the 52 HR pairs (P < 0.0001).

Finally, we tested for possible trade-offs between early- and late-life reproduction. We calculated the number of pups weaned during litters 1–2 and compared that with the number weaned during litters 4–12 or 5–12 (i.e., excluding litter 3). For all mice, the correlations were 0.385 (N = 67, 2-tailed P = 0.001) for litters 1–2 versus 4–12 and 0.358 (N = 54, P 0.008) for litters 1–2 versus 5–12. Separating by linetype, values were 0.449 (N = 37, P = 0.005) and 0.447 (N = 31, P = 0.012), respectively, for Control mice and 0.328 (N = 30, P = 0.077) and 0.257 (N = 23, P = 0.237) for HR mice. Thus, we found no evidence for a trade-off.

## Discussion

### Overview

We examined lifetime reproductive success and lifespan in mice sampled from generation 97 of a long-term, replicated artificial selection experiment for increased wheel-running behavior. We created 100 pairs of mice and found no statistically significant differences between the four selectively bred HR lines as compared with the four non-selected C lines for lifetime reproductive success (LRS) or any of its components (Fig. 1, Table 1). We also found no difference in average lifespan for the dams or sires. We did, however, find statistically significant differences among the HR lines and among the C lines for LRS and some of its components, and among the C lines for age at death for the male breeders (Fig. 1). We discuss these results in the context of life history theory and the potential trade-offs associated with selection for increased locomotor behavior.

### Lifetime Reproductive Success and Components of Darwinian Fitness

Selection on a given trait often engenders trade-offs with others because resources are limited or for other reasons, such as functional conflicts (Douhard et al. 2021; Garland et al. 2022; Husak and Lailvaux 2022; Shirley et al. 2022; Sadhir and Pontzer 2023; Josefson et al. 2024). The mechanisms that cause trade-offs should also be reflected as negative genetic correlations (Chang et al. 2024). However, we found no statistical evidence that Darwinian fitness has been reduced by artificial selection for high voluntary locomotor behavior. These results may simply indicate that the traits linked to high wheel-running behavior in the HR mice, such as higher endurance and VO_2_max (Meek et al. 2009; Schwartz et al. 2023), larger hearts (Schwartz and Garland 2024), and altered muscle properties (e.g., Castro et al. 2024), do not necessarily conflict with reproduction. Moreover, previous studies have not found notable differences in maternal behavior when these mice are housed without wheels (Girard et al. 2002; Keeney 2011; Hiramatsu et al. 2017; Schwartz et al. 2025).

Alternatively, or in addition, compensatory mechanisms may be at play, helping to maintain reproductive fitness despite changes in other traits that adversely affect LRS. For example, the HR mice might have adjusted their food intake, energy allocation, parental investment, or timing of reproduction in ways that balance the demands of reproduction with their presumably increased locomotor behavior in home cages (Copes et al. 2015; Hiramatsu and Garland 2018). Indeed, the average interbirth interval was longer in HR lines (P ~ 0.05: Table 1, Online Resource 3), perhaps because dams from the HR lines allocate more energy toward milk quantity or quality, or perhaps grooming of pups or keeping them warm, which potentially could delay ovulation via neuroendocrine or hormonal feedback mechanisms (Bittner et al. 2011; Chatzidaki et al. 2021) and hence birth of the next litter (Bittner et al. 2011). This might cause differences in postpartum recovery between HR and non-selected C lines. As well, the HR lines may have hormonal differences that affect the timing of ovulation or the maintenance of pregnancy. This could affect the period of time needed between births for the dam to recover, thus resulting in longer interbirth intervals. Such differences are speculative, but we did find at generation 44 that HR females spent significantly more time in the estrus stage of the estrous cycle compared to C females, along with a trend towards longer total cycle length in HR mice (Keeney 2011).

Nutritional factors may also contribute to the absence of life-history trade-offs. For example, Solon-Biet et al. (2015) showed that reproductive traits and lifespan in mice are optimized by different macronutrient balances. Mice in the present study had ad lib access to standardized food, as used during most of the protocol for the selection experiment, but they were never given the breeder diet (Harlan Teklad [Envigo] Lab Mouse Breeder Diet [S-2335] 7004) that, after 23 generations (Rhodes et al. 2003), we started administering to pregnant dams until the offspring were weaned to eliminate clostridial enteropathy (see Krugner-Higby et al. 2006). Thus, approximately 70 generations of the selection experiment experienced a dietary regimen that was somewhat different from that used in the present study. Reduced litter sizes in the present study could have been partly a function of this difference (see below).

A number of differences were detected among the replicate HR lines and among the C lines (Fig. 1, Online Resource 3). This variation shows that, unsurprisingly, factors beyond selection for high running behavior can influence reproductive characteristics. In the present experiment, random genetic drift will certainly have caused differences among replicate lines (e.g., Garland et al. 2002; Hillis and Garland 2023; Whitehead et al. 2023). Differences caused by drift may then lead to additional changes in related traits and promote the evolution of multiple solutions (Garland et al. 2011; Castro et al. 2024).

No previous study has housed the HR and C mice in breeding pairs to allow production of multiple litters (but see Krugner-Higby et al. 2006, who studied both first and second litters from a set of females). However, comparison of the sizes of the first litters at weaning (Online Resource 1) with studies at earlier generations indicates a reduction in average litter size for both HR and C lines (Krugner-Higby et al. 2006; Keeney 2011; Khan et al. 2024). The number of pups weaned for first litters in the present study averaged 9.07 ± 0.38 (LS Mean ± SE) for HR lines and 7.62 ± 0.80 for C lines (P = 0.1522). In contrast, litter sizes at weaning at generation 22 averaged 10.4 for both linetypes (Girard et al. 2002). [Larger first litter sizes for HR dams have been reported previously at other generations (Hiramatsu and Garland 2018)]. Such a reduction in a major component of fitness would be expected, given the unavoidable amount of inbreeding and, usually, inbreeding depression (Charlesworth and Willis 2009) that necessarily occurs in relatively small populations over many tens of generations (see Careau et al. 2013 on these lines).

### Lifespan and Mortality Rates

Relatively few studies have presented data on possible sex differences in lifespan in laboratory house mice. Under ad libitum feeding, as in the present study, sex differences in lifespan sometimes occur, but they vary by strain and with conditions (Mitchell et al. 2016; Cheng et al. 2019; e.g., see Baumann et al. 2019; Yuan et al. 2020; Garratt et al. 2020, 2021; Félix et al. 2025). In our study, mean lifespan did not differ statistically between HR and C lines for either mothers or fathers (Fig. 2, Table 1). Survival curves were generally similar among the four groups (Fig. 3), with a tendency for C dams to have an increase in instantaneous mortality rate beginning around 400–450 days of age (Fig. 4). The better fit of Gompertz models as compared with logistic models (see Sect. ‘‘Results’’) indicates that mortality rates did not plateau or decelerate in late life, which is generally consistent with existing data for laboratory mice (Gavrilova and Gavrilov 2015), and with two previous studies of the HR and C lines (Bronikowski et al. 2006; Vaanholt et al. 2010). Interestingly, we only found lifespan differences among the replicate lines for sires in the C lines. We found no statistically significant sex difference (and no sex-by-line interaction) in either C or HR lines.

Comparison of the present results with previous studies of the HR mice suggests that lifespan may also have decreased across generations. At generation 16, Bronikowski et al. (2006) reported lifespans of mice of both sexes housed individually (no reproduction allowed), with or without wheels. Approximately half of the surviving mice in each group were sacrificed at 84 weeks (588 days) of age for tissue harvesting, resulting in censored data (Robertson et al. 2011). Estimated median lifespans for all four groups housed without wheels (range = 760–880 days in their Table 1) were substantially longer than what we found (Table 2: range = 503–598 days). At generation 31, Vaanholt et al. (2010) recorded a median lifespan of 733 days for HR males housed without wheels. These results suggest that inbreeding depression may have reduced lifespan for both HR and C lines and/or that a lifetime of reproduction shortens lifespan (i.e., is costly with respect to lifespan).

Alternatively, or in addition, life-long reproductive opportunity (or even pair-housing per se [(e.g., see Garratt et al. 2020)] could have contributed to reduced lifespans. However, previous studies in laboratory mice suggest such effects are limited. Tarín et al. (2014) found no association between reproductive output and lifespan, whereas Mitchell et al. (2024) showed that reproduction increased mortality risk during active breeding but did not reduce subsequent post-reproductive survival among females that survived the breeding period. Likewise, Bhatnagar and Nielsen (2014) reported relatively modest effects of reproductive history up to one year on survival, despite substantial differences in maintenance energy expenditure.

### Relations Among Life History Traits at the Level of Individual Variation

Although physiological trade-offs are expected to exist among components of the life history (see Sect. ‘‘Introduction’’ and Garland et al. 2022), and have sometimes been reported for laboratory house mice (e.g., Garratt et al. 2020), we found few negative correlations when considering either the HR or C lines (Table 3). For both HR and C lines, lifetime reproductive success was significantly positively correlated with the dam’s age at birth of the last litter, the number of litters born and weaned, the ratio of the number of litters weaned divided by the number born (a likely indicator of parental care), and the average litter size at weaning (Online Resource 3).

We found no negative correlations between early- and late-life reproduction (pups weaned in litters 1–2 versus 4–12 or 5–12). Correlations between LRS and lifespan were non-significant in both linetypes. Both of these results are inconsistent with the idea of a cost of reproduction. The correlation between the dam’s and sire’s lifespan was non-significant for both the HR and C lines. Overall, our results are consistent with a recent meta-analysis of genetic correlations in a wide range of animal species, including a small number of mammals (Chang et al. 2024).

### Caveats, Conclusions, and Future Directions

One limitation of our study is that all mice were housed without wheels, which cannot fully capture the behavioral and physiological consequences of voluntary wheel-running behavior, especially in the HR lines. We did this because we were concerned about possible injury to pups and because space and monetary constraints did not permit us to maintain another set of 100 pairs mice with wheel access.

Although previous studies have found that singly housed HR mice are generally more active in home cages than are C mice (both sexes) when wheels are not available (Rhodes et al. 2001; Malisch et al. 2009; e.g., see Copes et al. 2015; Singleton and Garland 2019; Schwartz et al. 2025), these activity levels are well below the level of physical exertion and energy expenditure that occurs during wheel running (Rezende et al. 2009; Copes et al. 2015). On the other hand, the routine selection protocol includes wheel access for only six days, so housing mice without wheels reflects conditions experienced for most of the lifetime during a normal generation.

The controlled laboratory setting eliminates many environmental stressors [(e.g., food shortages or a need to work for food (Vaanholt et al. 2007; Zhao et al. 2018)] that could exacerbate potential trade-offs between locomotor behavior and reproduction under natural conditions. Future studies incorporating varying environmental conditions, including different degrees of food availability (e.g., see Thompson et al. 2024) and access to exercise wheels (e.g., see Schwartz et al. 2025), would provide further insights. Also, incorporating measures of milk quantity and quality could provide insight into maternal investment and potential effects on offspring performance.

We must also acknowledge that the statistical power to detect correlated responses to selection, including possible costs of reproduction and life history trade-offs, is generally low because the degrees of freedom for testing treatment effects are determined by the number of replicate lines, thus being 1 and 6 in the present experiment. Moreover, simulations show that the nested mixed model analysis, necessitated by the experimental design, often has reduced Type I error rates (e.g., see simulations in Castro et al. 2021). Nonetheless, numerous differences between the HR and C lines have been detected in previous studies (see Sect. ‘‘Introduction’’).

Those caveats aside, the absence of significant differences in LRS and lifespan between HR and C lines challenges the traditional view of energy allocation theory, which posits that increased energy expenditure on physical activity should detract from other fitness components, such as reproduction and somatic maintenance. Our findings suggest that HR mice may possess compensatory mechanisms (including simply increased food consumption) that allow them to maintain "normal" reproduction and longevity, despite increased levels of locomotor behavior. Future studies could test for such mechanisms, which could include enhanced metabolic efficiency, better stress resilience, or other physiological or behavioral adaptations that mitigate the expected trade-offs.

## Supplementary Information

Below is the link to the electronic supplementary material.Supplementary file1 (XLSX 9 KB)−Descriptive statistics for all traitsSupplementary file2 (XLSX 133 KB)−Frequency of concurrent pregnancy and lactation Concurrent_Pregnancy_and_Lactation_CPL_Litter_Overlap_1.xlsxSupplementary file3 (XLSX 46 KB)−Full version of Table 1, with all of the line comparisons SAS_Results_Linetype_21.xlsx

## Data Availability

Data and code are available at Figshare https://doi.org/10.6084/m9.figshare.30223621.
